# Supplementary material for: Mental Health and Quality of Life among Patients with Cancer during the SARS-CoV-2 Pandemic: Results from the Longitudinal ONCOVID Survey Study
Source: Cancers (Basel). 2022 Feb 21;14(4):1093. doi: 10.3390/cancers14041093 (PMC8869927; doi:10.3390/cancers14041093)
Supplement: Supplementary file 1 [file cancers-14-01093-s001.zip › cancers-1528688-supplementary.docx]

Supplementary Material

Mental Health and Quality of Life among Patients with Cancer during the SARS-CoV-2 Pandemic: Results from the
Longitudinal ONCOVID Survey Study

**Emiel A. De Jaeghere ^1,2,^*, Heini Kanervo ^1^, Roos Colman ^3^, Wim Schrauwen ^4^, Paulien West ^5^, Nele Vandemaele ^5^, Aglaja De Pauw ^1^, Celine Jacobs ^1^, Ingeborg Hilderson ^1^, Michael Saerens ^1^, Nora Sundahl ^2,6^,
Katrien Vandecasteele ^2,6^, Eline Naert ^1,2^, Lore Lapeire ^1,2^, Vibeke Kruse ^2,7^, Sylvie Rottey ^1,2^, Gilbert Lemmens ^8^
and Hannelore G. Denys ^1,2^**

^1^ Medical Oncology, Ghent University Hospital, 9000 Ghent, Belgium; [heini.kanervo@uzgent.be](mailto:heini.kanervo@uzgent.be) (H.K.);
[aglaja.depauw@ugent.be](mailto:aglaja.depauw@ugent.be) (A.D.P.); [celine.jacobs@uzgent.be](mailto:celine.jacobs@uzgent.be) (C.J.); [ingeborg.hilderson@uzgent.be](mailto:ingeborg.hilderson@uzgent.be) (I.H.);
[michael.saerens@ugent.be](mailto:michael.saerens@ugent.be) (M.S.); [eline.naert@ugent.be](mailto:eline.naert@ugent.be) (E.N.); [lore.lapeire@uzgent.be](mailto:lore.lapeire@uzgent.be) (L.L.);
[sylvie.rottey@ugent.be](mailto:sylvie.rottey@ugent.be) (S.R.); [hannelore.denys@ugent.be](mailto:hannelore.denys@ugent.be) (H.G.D.)

^2^ Cancer Research Institute Ghent (CRIG), 9000 Ghent, Belgium; [nora.sundahl@ugent.be](mailto:nora.sundahl@ugent.be) (N.S.);
[katrien.vandecasteele@ugent.be](mailto:katrien.vandecasteele@ugent.be) (K.V.); [vibeke.kruse@ugent.be](mailto:vibeke.kruse@ugent.be) (V.K.)

^3^ Biostatistics Unit, Faculty of Medicine and Life Sciences, Ghent University, 9000 Ghent, Belgium;
[roos.colman@ugent.be](mailto:roos.colman@ugent.be)

^4^ Medical Psychology, Ghent University Hospital, 9000 Ghent, Belgium; [wim.schrauwen@uzgent.be](mailto:wim.schrauwen@uzgent.be)

^5^ Faculty of Medicine and Life Sciences, Ghent University, 9000 Ghent, Belgium; [paulien.west@ugent.be](mailto:paulien.west@ugent.be) (P.W.);
[nele.vandemaele@ugent.be](mailto:nele.vandemaele@ugent.be) (N.V.)

^6^ Radiotherapy, Ghent University Hospital, 9000 Ghent, Belgium

Medical Oncology, General Hospital Sint-Nikolaas, 9100 Sint-Niklaas, Belgium

^8^ Psychiatry, Ghent University Hospital, 9000 Ghent, Belgium; [gilbert.lemmens@ugent.be](mailto:gilbert.lemmens@ugent.be)

***** Correspondence: emiel.dejaeghere@ugent.be; Tel.: +32-9-332-26-92


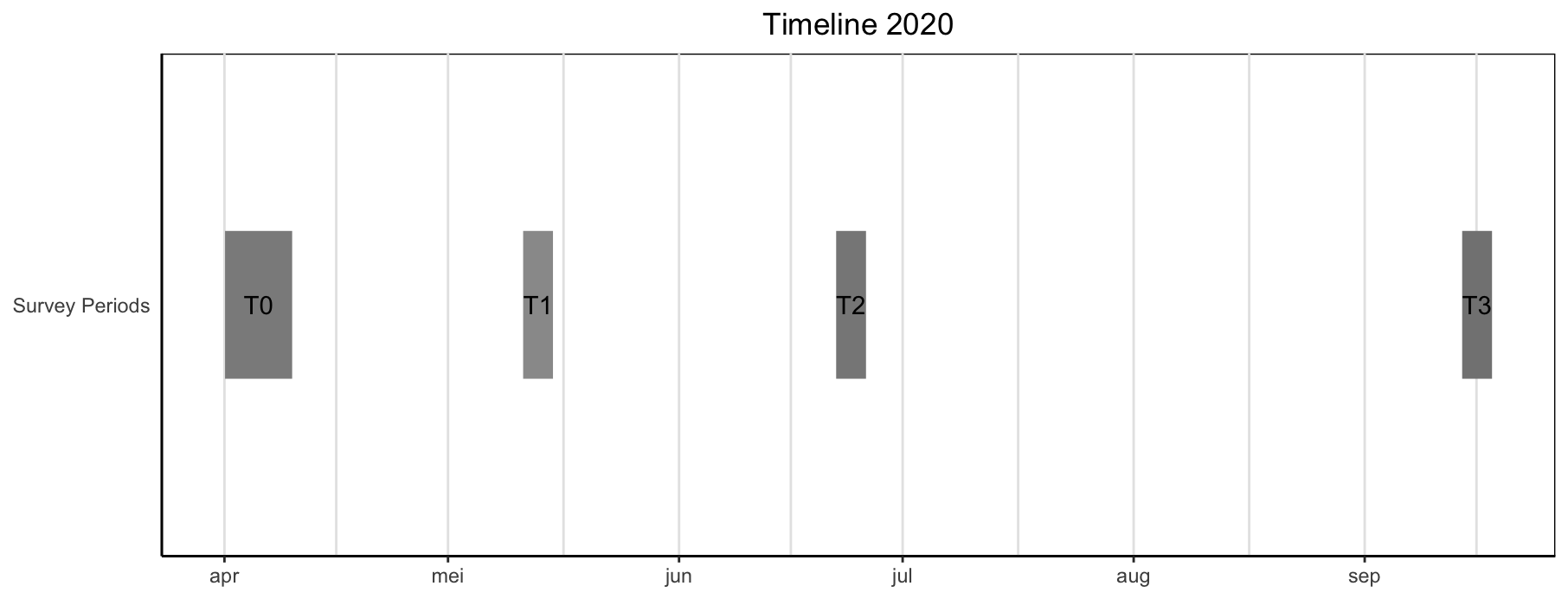


**Figure S1.** Study timeline.

**Figure S2.** Correlation matrix.

Pearson correlation coefficients between the different scores.

**Table S1.** Summary of descriptive statistics of scores at T0 and minimal important difference.

| **Variable** | **Mean (SD) at Baseline** | **MID** |
| --- | --- | --- |
| **CPDI** |  |  |
| Peritraumatic distress | 26.86 (13.08) | 6.54 |
| **DASS-21** |  |  |
| Depression | 3.14 (3.69) | 1.85 |
| Anxiety | 2.37 (2.88) | 1.44 |
| Stress | 3.47 (3.63) | 1.82 |
| **WHOQOL-BREF** |  |  |
| Physical health | 63.51 (18.83) | 9.42 |
| Psychological health | 64.11 (14.88) | 7.44 |
| Social relationships | 69.43 (16.08) | 8.04 |
| Environmental health | 75.40 (13.94) | 6.96 |
| Overall QOL and general health | 6.59 (1.59) | 0.80 |

MID—minimal important difference; QOL—quality of life; CPDI—COVID-19 Peritraumatic Distress Index; SD—standard deviation

**Table S2.** Corresponding numerical values Figures 2 and 3.

|  | **Survey Period** | | | |
| --- | --- | --- | --- | --- |
| **Variable** | **T0** | **T1** | **T2** | **T3** |
| **Peritraumatic distress** |  |  |  |  |
| Normal | 60.3% | 62.9% | 64.5% | 65.9% |
| Moderate | 35.3% | 31.5% | 32.4% | 30.5% |
| Severe | 4.3% | 5.6% | 3.1% | 3.7% |
| **Depression** |  |  |  |  |
| Normal | 72.4% | 67.1% | 66.4% | 68.5% |
| Mild | 13.5% | 10.0% | 12.2% | 13.7% |
| Moderate | 7.5% | 16.9% | 12.2% | 12.4% |
| Severe | 4.2% | 2.5% | 5.9% | 3.3% |
| Extremely severe | 2.4% | 3.5% | 3.2% | 2.1% |
| **Anxiety** |  |  |  |  |
| Normal | 75.1% | 67.7% | 67.4% | 72.2% |
| Mild | 12.3% | 15.7% | 12.2% | 12.0% |
| Moderate | 6.6% | 8.5% | 10.8% | 8.3% |
| Severe | 2.4% | 3.5% | 4.9% | 4.2% |
| Extremely severe | 3.6% | 4.7% | 4.9% | 3.3% |
| **Stress** |  |  |  |  |
| Normal | 88.6% | 85.0% | 84.3% | 85.5% |
| Mild | 3.3% | 6.3% | 8.0% | 5.4% |
| Moderate | 4.8% | 5.0% | 4.2% | 5.8% |
| Severe | 3.0% | 3.5% | 3.2% | 2.9% |
| Extremely severe | 0.3% | 0.3% | 0.4% | 0.4% |

Percentages may not total 100% due to rounding.

**Table S3.** Corresponding datapoints Figure 4.

|  | **Survey Period** | | | |
| --- | --- | --- | --- | --- |
| **Variable** | **T0** | **T1** | **T2** | **T3** |
| **Mental health** |  |  |  |  |
| Peritraumatic distress  (0–100) | 26.94 (25.58 to 28.30) | 25.77 (24.32 to 27.22) | 24.28 (22.84 to 25.73) | 24.89 (23.35 to 26.43) |
| Depression (0–21) | 3.27 (2.87 to 3.67) | 3.68 (3.26 to 4.11) | 3.61 (3.16 to 4.06) | 3.78 (3.33 to 4.23) |
| Anxiety (0–21) | 2.42 (2.11 to 2.74) | 2.90 (2.57 to 3.24) | 2.86 (2.51 to 3.20) | 2.87 (2.50 to 3.23) |
| Stress (0–21) | 3.53 (3.15 to 3.92) | 3.81 (3.41 to 4.22) | 3.89 (3.49 to 4.29) | 4.03 (3.59 to 4.47 |
| **Quality of life** |  |  |  |  |
| Physical health (0–100) | 63.50 (61.55 to 65.46) | 62.10 (60.08 to 64.13) | 61.86 (59.76 to 63.96) | 61.39 (59.16 to 63.61) |
| Psychological health (0–100) | 64.11 (62.54 to 65.67) | 62.63 (60.95 to 64.31) | 63.30 (61.61 to 65.00) | 62.52 (60.68 to 64.37) |
| Social relationships (0–100) | 69.19 (67.50 to 70.88) | 67.06 (65.31 to 68.81) | 67.11 (65.31 to 68.92) | 65.44 (63.32 to 67.55) |
| Environment (0–100) | 75.39 (73.95 to 76.83) | 74.54 (73.02 to 76.06) | 74.67 (73.09 to 76.25) | 74.05 (72.35 to 75.74) |
| Overall QOL and general health (0–10) | 6.58 (6.42 to 6.74) | 6.47 (6.30 to 6.64) | 6.58 (6.34 to 6.81) | 6.69 (6.02 to 7.36) |

Data are expressed as mean with 95% confidence interval (CI). QOL—quality of life.

**Table S4.** Bivariate associations between characteristics of the participants and mental health scores.

|  | **Mental Health Scores** | | | | | | | |
| --- | --- | --- | --- | --- | --- | --- | --- | --- |
|  | **CPDI** | | **DASS-21** | | | | | |
| **Variable** | **Peritraumatic distress**  **(0–100)** | ***p* Value** | **Depression**  **(0–21)** | ***p* Value** | **Anxiety**  **(0–21)** | ***p* Value** | **Stress**  **(0–21)** | ***p* Value** |
| **Age** | −0.03 (−0.13 to 0.07) | 0.571 | −0.01 (−0.04 to 0.02) | 0.616 | 0.01 (−0.01 to 0.03) | 0.317 | −0.01 (−0.04 to 0.01) | 0.289 |
| **Gender** |  | 0.091 |  | 0.839 |  | 0.001 |  | 0.950 |
| Female | 26.16 (24.64 to 27.69) |  | 3.56 (3.11 to 4.01) |  | 2.47 (2.13 to 2.81) |  | 3.83 (3.41 to 4.24) |  |
| Male | 23.72 (21.31 to 26.13) |  | 3.65 (2.95 to 4.35) |  | 3.48 (2.95 to 4.01) |  | 3.80 (3.15 to 4.45) |  |
| **Educational level** |  | 0.117 |  | 0.701 |  | 0.068 |  | 0.684 |
| Fundamental or secondary | 26.41 (24.58 to 28.24) |  | 3.61 (3.07 to 4.15) |  | 3.02 (2.60 to 3.43) |  | 3.87 (3.38 to 4.36) |  |
| Higher education | 24.36 (22.53 to 26.19) |  | 3.46 (2.93 to 4.00) |  | 2.48 (2.07 to 2.89) |  | 3.73 (3.24 to 4.22) |  |
| **Marital status** |  | 0.466 |  | 0.539 |  | 0.622 |  | 0.872 |
| Single or never married | 24.31 (20.43 to 28.20) |  | 3.77 (2.63 to 4.90) |  | 2.99 (2.13 to 3.85) |  | 3.58 (2.53 to 4.63) |  |
| Married or living as married | 25.33 (23.81 to 26.85) |  | 3.46 (3.02 to 3.91) |  | 2.69 (2.35 to 3.03) |  | 3.88 (3.46 to 4.29) |  |
| Divorced, separated, or widowed | 27.24 (24.00 to 30.47) |  | 4.03 (3.08 to 4.98) |  | 3.02 (2.29 to 3.75) |  | 3.87 (2.99 to 4.75) |  |
| **Household composition** |  | 0.555 |  | 0.102 |  | 0.032 |  | 0.938 |
| Living with others | 25.31 (23.86 to 26.76) |  | 3.45 (3.02 to 3.87) |  | 2.63 (2.30 to 2.95) |  | 3.84 (3.45 to 4.23) |  |
| Living alone | 26.30 (23.33 to 29.28) |  | 4.25 (3.38 to 5.12) |  | 3.42 (2.77 to 4.08) |  | 3.88 (3.08 to 4.68) |  |
| **Cancer type** |  | 0.430 |  | 0.379 |  | 0.080 |  | 0.846 |
| Breast | 26.55 (24.60 to 28.50) |  | 3.59 (3.02 to 4.17) |  | 2.64 (2.20 to 3.07) |  | 3.88 (3.35 to 4.41) |  |
| Genitourinary | 24.30 (21.05 to 27.54) |  | 4.06 (3.12 to 5.01) |  | 3.34 (2.63 to 4.05) |  | 3.97 (3.09 to 4.84) |  |
| Gynecological | 25.98 (22.58 to 29.38) |  | 3.35 (2.36 to 4.34) |  | 2.31 (1.56 to 3.05) |  | 4.07 (3.15 to 4.99) |  |
| Melanoma | 22.10 (18.56 to 25.65) |  | 2.64 (1.61 to 3.67) |  | 2.21 (1.43 to 2.99) |  | 3.37 (2.41 to 4.32) |  |
| Head and neck | 25.74 (20.50 to 30.98) |  | 3.79 (2.23 to 5.35) |  | 3.91 (2.72 to 5.10) |  | 3.23 (1.77 to 4.68) |  |
| Soft tissue and bone | 27.88 (21.60 to 34.17) |  | 4.69 (2.92 to 6.47) |  | 3.57 (2.23 to 4.90) |  | 4.36 (2.71 to 6.00) |  |
| Other | 24.47 (17.47 to 31.46) |  | 4.20 (2.17 to 6.23) |  | 2.78 (1.26 to 4.30) |  | 3.28 (1.40 to 5.16) |  |
| **Therapy intention** |  | 0.926 |  | 0.432 |  | 0.600 |  | 0.757 |
| Palliative | 25.50 (24.04 to 26.97) |  | 3.67 (3.24 to 4.09) |  | 2.80 (2.47 to 3.13) |  | 3.85 (3.45 to 4.24) |  |
| Curative | 25.36 (22.58 to 28.13) |  | 3.30 (2.49 to 4.11) |  | 2.62 (2.00 to 3.23) |  | 3.71 (2.97 to 4.46) |  |
| **Estimated life expectancy** |  | 0.178 |  | 0.071 |  | <0.001 |  | 0.476 |
| >1 year | 25.00 (23.53 to 26.47) |  | 3.40 (2.97 to 3.83) |  | 2.49 (2.17 to 2.82) |  | 3.75 (3.35 to 4.15) |  |
| ≤1 year | 27.08 (24.40 to 29.77) |  | 4.22 (3.43 to 5.00) |  | 3.68 (3.09 to 4.28) |  | 4.05 (3.32 to 4.77) |  |
| **Therapy change** |  | 0.387 |  | 0.341 |  | 0.100 |  | 0.166 |
| Yes | 23.92 (20.16 to 27.67) |  | 3.08 (1.98 to 4.19) |  | 2.10 (1.27 to 2.94) |  | 3.14 (2.13 to 4.16) |  |
| No | 25.68 (24.30 to 27.06) |  | 3.65 (3.25 to 4.05) |  | 2.85 (2.54 to 3.16) |  | 3.91 (3.54 to 4.28) |  |

Data are mean (95% CI). For age, data are unadjusted regression coefficients (95% CI) for a one-year increase.

**Table S5.** Bivariate associations between characteristics of participants and quality-of-life scores.

|  | **QOL Scores** | | | | | | | | | |
| --- | --- | --- | --- | --- | --- | --- | --- | --- | --- | --- |
|  | **WHOQOL-BREF** | | | | | | | | | |
| **Variable** | **Physical health**  **(0–100)** | ***p* Value** | **Psychological health**  **(0–100)** | ***p* Value** | **Social relationships**  **(0–100)** | ***p V*alue** | **Environment**  **(0–100)** | ***p* Value** | **Overall QOL**  **(0–10)** | ***p* Value** |
| **Age** | 0.10 (−0.05 to 0.24) | 0.182 | 0.14 (0.02 to 0.26) | 0.018 | 0.02 (−0.11 to 0.14) | 0.789 | −0.01 (−0.12 to 0.10) | 0.842 | 0.01 (−0.01 to 0.02) | 0.334 |
| **Gender** |  | 0.974 |  | 0.826 |  | <.001 |  | 0.193 |  | 0.829 |
| Female | 62.20 (60.03 to 64.36) |  | 63.03 (61.27 to 64.80) |  | 69.42 (67.59 to 71.25) |  | 75.23 (73.60 to 76.85) |  | 6.59 (6.33 to 6.85) |  |
| Male | 62.26 (58.83 to 65.70) |  | 63.40 (60.61 to 66.20) |  | 61.55 (58.67 to 64.42) |  | 73.23 (70.67 to 75.80) |  | 6.55 (6.22 to 6.89) |  |
| **Educational level** |  | 0.528 |  | 0.673 |  | 0.445 |  | <0.001 |  | 0.968 |
| Fundamental or secondary | 61.71 (59.09 to 64.32) |  | 62.82 (60.70 to 64.95) |  | 66.84 (64.61 to 69.08) |  | 72.41 (70.49 to 74.34) |  | 6.58 (6.29 to 6.88) |  |
| Higher education | 62.89 (60.28 to 65.50) |  | 63.46 (61.34 to 65.59) |  | 68.05 (65.82 to 70.28) |  | 77.21 (75.29 to 79.14) |  | 6.59 (6.30 to 6.88) |  |
| **Marital status** |  | 0.040*^i^* |  | 0.332 |  | 0.080 |  | 0.004*^ii^* |  | 0.226 |
| Single or never married | 61.84 (56.35 to 67.32) |  | 63.59 (59.10 to 68.07) |  | 65.29 (60.59 to 69.99) |  | 75.83 (71.75 to 79.91) |  | 6.56 (6.08 to 7.04) |  |
| Married or living as married | 63.37 (61.24 to 65.51) |  | 63.56 (61.81 to 65.31) |  | 68.35 (66.50 to 70.20) |  | 75.66 (74.07 to 77.26) |  | 6.65 (6.39 to 6.90) |  |
| Divorced, separated, or widowed | 56.88 (52.31 to 61.45) |  | 60.49 (56.75 to 64.23) |  | 63.82 (59.89 to 67.74) |  | 69.36 (65.96 to 72.76) |  | 6.29 (5.88 to 6.71) |  |
| **Household composition** |  | 0.240 |  | 0.377 |  | 0.020 |  | 0.151 |  | 0.303 |
| Living with others | 62.56 (60.51 to 64.60) |  | 63.30 (61.63 to 64.97) |  | 68.02 (66.26 to 69.78) |  | 75.10 (73.56 to 76.63) |  | 6.54 (6.37 to 6.71) |  |
| Living alone | 59.76 (55.55 to 63.97) |  | 61.59 (58.16 to 65.02) |  | 63.33 (59.74 to 66.92) |  | 72.55 (69.40 to 75.70) |  | 6.34 (5.99 to 6.69) |  |
| **Cancer type** |  | 0.075 |  | 0.543 |  | <0.001*^iii^* |  | 0.033 *^iv^* |  | 0.021*^v^* |
| Breast | 62.42 (59.68 to 65.16) |  | 64.20 (61.95 to 66.46) |  | 69.97 (67.65 to 72.29) |  | 76.58 (74.53 to 78.62) |  | 6.64 (6.35 to 6.94) |  |
| Genitourinary | 60.45 (55.89 to 65.00) |  | 60.99 (57.24 to 64.74) |  | 61.19 (57.37 to 65.02) |  | 71.75 (68.36 to 75.15) |  | 6.49 (6.08 to 6.90) |  |
| Gynecological | 64.51 (59.72 to 69.30) |  | 63.82 (59.88 to 67.75) |  | 69.54 (65.52 to 73.55) |  | 74.95 (71.38 to 78.51) |  | 6.53 (6.11 to 6.95) |  |
| Melanoma | 66.03 (61.04 to 71.02) |  | 64.01 (59.91 to 68.11) |  | 65.63 (61.43 to 69.82) |  | 75.43 (71.72 to 79.15) |  | 7.07 (6.63 to 7.51) |  |
| Head and neck | 61.40 (53.96 to 68.83) |  | 61.94 (55.84 to 68.04) |  | 64.73 (58.47 to 71.00) |  | 69.56 (64.04 to 75.08) |  | 6.09 (5.47 to 6.72) |  |
| Soft tissue and bone | 49.85 (41.02 to 58.69) |  | 57.41 (50.15 to 64.68) |  | 56.89 (49.48 to 64.30) |  | 68.16 (61.59 to 74.73) |  | 5.76 (5.04 to 6.49) |  |
| Other | 60.04 (50.19 to 69.90) |  | 62.33 (54.25 to 70.42) |  | 72.59 (64.32 to 80.86) |  | 76.32 (68.99 to 83.65) |  | 6.33 (5.52 to 7.13) |  |
| **Therapy intention** |  | 0.897 |  | 0.347 |  | 0.017 |  | 0.887 |  | 0.243 |
| Palliative | 62.15 (60.08 to 64.22) |  | 63.51 (61.82 to 65.20) |  | 66.21 (64.43 to 68.00) |  | 74.61 (73.05 to 76.17) |  | 6.54 (6.28 to 6.79) |  |
| Curative | 62.45 (58.50 to 66.39) |  | 61.78 (58.57 to 64.99) |  | 70.81 (67.45 to 74.17) |  | 74.85 (71.91 to 77.80) |  | 6.74 (6.38 to 7.11) |  |
| **Estimated life expectancy** |  | 0.001 |  | 0.047 |  | 0.005 |  | 0.015 |  | <0.001 |
| >1 year | 63.82 (61.77 to 65.87) |  | 63.94 (62.25 to 65.64) |  | 68.39 (66.60 to 70.19) |  | 75.57 (74.02 to 77.13) |  | 6.75 (6.50 to 7.01) |  |
| ≤1 year | 56.75 (52.97 to 60.52) |  | 60.39 (57.29 to 63.49) |  | 63.14 (59.89 to 66.39) |  | 71.59 (68.75 to 74.42) |  | 5.98 (5.62 to 6.33) |  |
| **Therapy change** |  | 0.035 |  | 0.164 |  | 0.097 |  | 0.136 |  | 0.090 |
| Yes | 67.57 (62.26 to 72.88) |  | 66.03 (61.69 to 70.36) |  | 70.82 (66.25 to 75.38) |  | 77.50 (73.52 to 81.48) |  | 6.92 (6.46 to 7.39) |  |
| No | 61.51 (59.57 to 63.45) |  | 62.76 (61.17 to 64.35) |  | 66.72 (65.02 to 68.42) |  | 74.29 (72.82 to 75.76) |  | 6.54 (6.29 to 6.79) |  |

Data are mean (95% confidence interval [CI]). For age, data are unadjusted regression coefficients (95% CI) for a one-year increase. Bonferroni-corrected post-hoc thresholds were applied after obtaining significant results: *i* significant differences in pairwise comparisons (threshold *p* < 0.01667): married or living as married vs. divorced, separated, or widowed (estimated difference [ED], 6.49 [1.37 to 11.62]; *P* = 0.011); *ii* significant differences in pairwise comparisons (threshold *p* < 0.01667): single or never married vs. divorced, separated, or widowed (ED, 6.47 [1.08 to 11.87]; *p* = 0.01659); and married or living as married vs. divorced, separated, or widowed (ED, 6.30 [2.50 to 10.10]; *p* = 0.001); *iii* significant differences in pairwise comparisons (threshold *p* < 0.0024): breast vs. genitourinary (ED, 8.78 [4.25 to 13.30]; *p* < 0.001); and breast vs. soft tissue and bone (ED, 13.08 [5.19 to 20.97]; *p* < 0.001); *iv* significant differences in pairwise comparisons (threshold *p* < 0.0024): none; *v* significant differences in pairwise comparisons (threshold *p* < 0.0024): melanoma vs. soft tissue and bone (ED, 1.31 [0.49 to 2.12]; *p* = 0.001). QOL—quality of life.
